# Age-Period-Cohort Analysis of Type 2 Diabetes Mortality Attributable to Particulate Matter Pollution in China and the U.S.

**DOI:** 10.1155/2020/1243947

**Published:** 2020-06-11

**Authors:** Xiaoxue Liu, Maigeng Zhou, Chuanhua Yu, Zhi-Jiang Zhang

**Affiliations:** ^1^Department of Preventive Medicine, School of Health Sciences, Wuhan University, Wuhan 430071, China; ^2^National Center for Chronic and Noncommunicable Disease Control and Prevention, Chinese Center for Disease Control and Prevention, Nanwei Road 27, Xicheng District, Beijing 100050, China

## Abstract

**Aim:**

We aimed to assess and compare secular trends in type 2 diabetes mortality attributable to particulate matter pollution in China and U.S.

**Methods:**

We performed an age-period-cohort (APC) analysis to estimate the independent effects of age, period, and cohort on mortality of type 2 diabetes attributable to particulate matter pollution. We collected age-standardized and age-specific mortality rates (1990-2017) from the Global Burden of Disease 2017 Study for China and the U.S.

**Results:**

During the period 1990-2017, the age-standardized mortality rates of type 2 diabetes attributable to particulate matter pollution in China showed a general increasing trend, while that in U.S. showed an increase before 2002 and subsequently a decrease. The age effect increased markedly in China compared with the U.S. The period effect showed a substantially increase in China while that in the U.S. increased during 1990-2007 and tended to be stable during 2007-2017. The cohort effect peaked in birth cohort born in 1902–1906 in both China and U.S. and declined consistently in the cohort born in 1992-1996.

**Conclusions:**

The age-standardized mortality rates of type 2 diabetes attributable to particulate matter pollution, the age, and period effect in China have been increasing in both sexes from 1990 to 2017. The overall mortality in the U.S. began to decrease since 2003, and the period effect showed a tendency to stabilize. Consequently, it is necessary to educate the nation with the correct knowledge and adopting policies on pollutant emission and techniques to reduce air pollution in China.

## 1. Introduction

Diabetes is one of the largest epidemics the world faces in the 21st century [1–3]. The age-standardized prevalence of diabetes in adults worldwide has increased since 1980, and diabetes burden has increased faster in low- and middle-income countries than in high-income countries, and gender difference exists in diabetes prevalence [4]. In China alone, there were an estimated 3,338,131 new cases and 153,184 deaths of diabetes in 2017, accounting for 14.55% and 11.18% of all new cases and all deaths worldwide, respectively [5, 6]. Previous study showed diabetes mortality significantly increased in men in China through 1990 to 2017, and women showed no significant changes [7]. However, the specific reasons for the increasing trend in diabetes mortality remain unclear, and etiology studies are needed to be conducted to explore the underlying causes of this trend.

Air pollution has an increased association with risk of type 2 diabetes [8, 9], and particulate matter air pollution is prevalent in China. The association between diabetes and air pollution is being concerned. Type 2 diabetes accounts for around 90% of diabetes types. The global toll of diabetes attributable to PM_2·5_ air pollution is significant [10]. A previous study reported that exposure to particulate matter pollution has been suggested as a contributing factor to type 2 diabetes incidence and progression [11–13]. Analysis of type 2 diabetes attributable to particulate matter pollution would provide a better understanding of the epidemiology of diabetes, identify endemic areas, and further contribute to the global and national discussions on the hazardous effect of air pollution on diabetes. Thus, in this study, we aimed to further estimate the trend of type 2 diabetes mortality attributable to particulate matter pollution to throw light on policy making regarding diabetes prevention and intervention, using an age-period-cohort study design.

## 2. Materials and Methods

### 2.1. Data Source

The age-standardized mortality rates of type 2 diabetes attributable to particulate matter pollution were obtained from GBD 2017, and these data for all ages were age-standardized by the GBD 2017 global age-standard population [6]. The mortality database is composed of vital registration (VR), verbal autopsy (VA), registry, survey, police, and surveillance data, and statistical modelling tools was used, including the Cause of Death Ensemble model (CODEm), to generate cause fractions and cause-specific death rates for each location, year, age, and sex [14]. The original data of diabetes mortality in China population was mainly from the Cause of Death Reporting System of the Chinese Center for Disease Control and Prevention (CDC), Disease Surveillance Points (DSPs), and the Maternal and Child Surveillance System, which are considered to be nationally representative [15]. In the following analysis, data of the groups under 25 years old was excluded as the type of diabetes diagnosed most commonly in these age groups is rare, and the groups above 95 years old were also excluded. For comparison, we also collected mortality data of type 2 diabetes for the U.S. during the period 1990-2017.

Based on comparative risk assessment from the GBD 2017 study, particulate matter pollution includes ambient particulate matter pollution (population-weighted annual average daily exposure to outdoor air concentrations of particulate matter with an aerodynamic diameter of ≤2·5 *μ*m (PM_2·5_) in a cubic meter of air, measured in *μ*g/m^3^), and household air pollution from solid fuels (individual exposure to PM_2·5_ due to the use of solid cooking fuel). The data used to estimate exposure to ambient air pollution is drawn from multiple sources, including satellite observations of aerosols in the atmosphere, ground measurements, chemical transport model simulations, population estimates, and land-use data. Data estimated for household air pollution were extracted from the standard multicountry survey series such as Demographic and Health Surveys (DHS), Living Standards Measurement Surveys (LSMS), Multiple Indicator Cluster Surveys (MICS), and World Health Surveys (WHS), as well as country-specific survey series such as Kenya Welfare Monitoring Survey and South Africa General Household Survey. The GBD 2017 study estimated the long-term exposure to ambient particulate matter pollution for both ambient and household air pollution by combining satellite data with a chemical transport model and land use information and calibrated satellite measurements to ground measurements using the Data Integration Model for Air Quality. The estimates on exposure to particulate matter pollution ere based on a whole population for both high and less polluted areas in China.

### 2.2. Age-Period-Cohort (APC) Analysis

Age reflects variations in vital rates, based on that mortality risk increases with the process of ageing. Period effects represent influential factors, including complex sets of historical events and environmental factors, that simultaneously affect all age groups. Cohort effects represent variations across groups of individuals born in the same year or years. The three effects influence morbidity and mortality risks in specific ways [14]. APC analysis offers an important way to decompose these trends in diabetes mortality and an opportunity to give hypotheses regarding effective measures to control and prevent diabetes in China. To estimate the unique set for every age, period, and cohort effect on mortality of diseases, the intrinsic estimator (IE) method associated with the APC model was proposed by Yang and Fu to decompose three temporal trends and provides unbiased and relatively efficient estimation results [16, 17]. In this study, we fitted common three-factor models, including APC models solved with a conventional constrained Poisson log-linear model estimator (APC-C) and IE method, and chose the best-fitting models. Goodness-of-fit statistics and the best-fitting model criteria were models with the same df (degree of freedom), smaller deviance values denoting a higher degree of fit, and smaller values for Akaike's Information Criterion (AIC) and Bayesian information criterion (BIC) with parameter penalty terms denoting a better fit. In this study, we selected APC (IE) models for analyzing diabetes data, as the results indicated that the IE method provided a best fit compared with the constrained Poisson log-linear model (see Supplementary Table A). APC model could be expressed as

(1) $$\begin{array}{c} Y_{j}=\mu +\alpha \;{\text{age}}_{j}+\beta \;{\text{period}}_{j}+\gamma \;{\text{cohort}}_{j}+\varepsilon_{i}, \end{array}$$

where *Y*_*j*_ denoted the response variable—the net effect on type 2 diabetes mortality for groups *j*,*α*,*β*, and *γ* denoted the coefficient of age, period, and cohort of the APC model, respectively, and *μ* denoted the intercept of the model. *ε*_*i*_ denoted the residual of the APC model.

**Figure 1:**
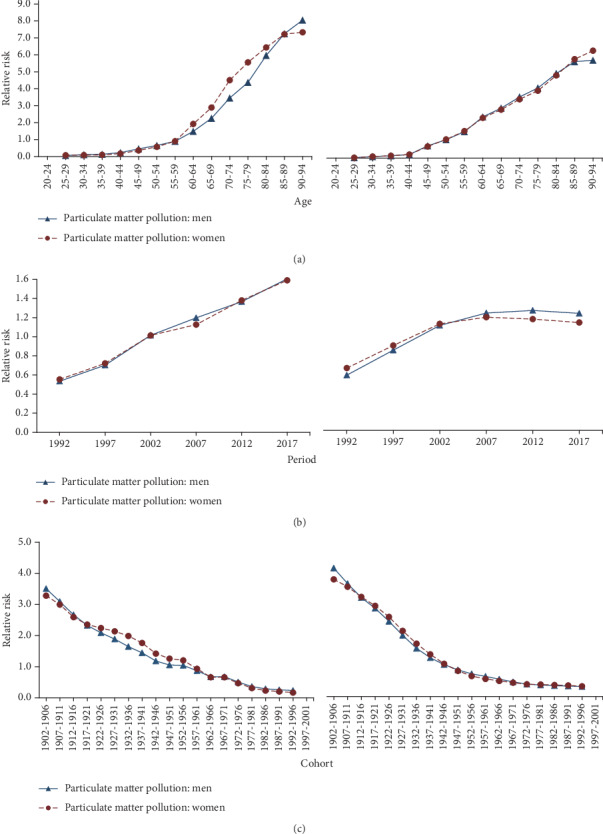
Type 2 diabetes mortality attributable to particulate matter pollution relative risks due to (a) age, (b) period, and (c) cohort effects. The left graph is China, and the right is the U.S.

In this analysis, the age-specific rates were appropriately recorded into successive 5-year age groups (25–29, 30–34, ..., 90–94), consecutive 5-year periods from 1990 to 2017, and correspondingly consecutive 5-year birth cohort groups (1902-1906, 1907-1911,…, 1992-1996) to estimate net age, period, and cohort effects. The APC with IE analysis presented estimated coefficients for the age, period, and cohort effects (see Supplementary Table B), and then, these coefficients were calculated to their exponential value (exp(coef.) = *e*^coef.^) that denoted the mortality relative risk (RR) of a particular age, period, or birth cohort relative to each average level [18] (see Supplementary Table C and D).  Figure 1 is plotted based on Supplementary Table C and D. The APC model was performed through the Stata 12.0 software (StataCorp, College Station, TX, USA).

## 3. Results

### 3.1. Descriptive Analysis of Type 2 Diabetes Mortality Attributable to Particulate Matter Pollution in China and U.S

**Figure 2:**
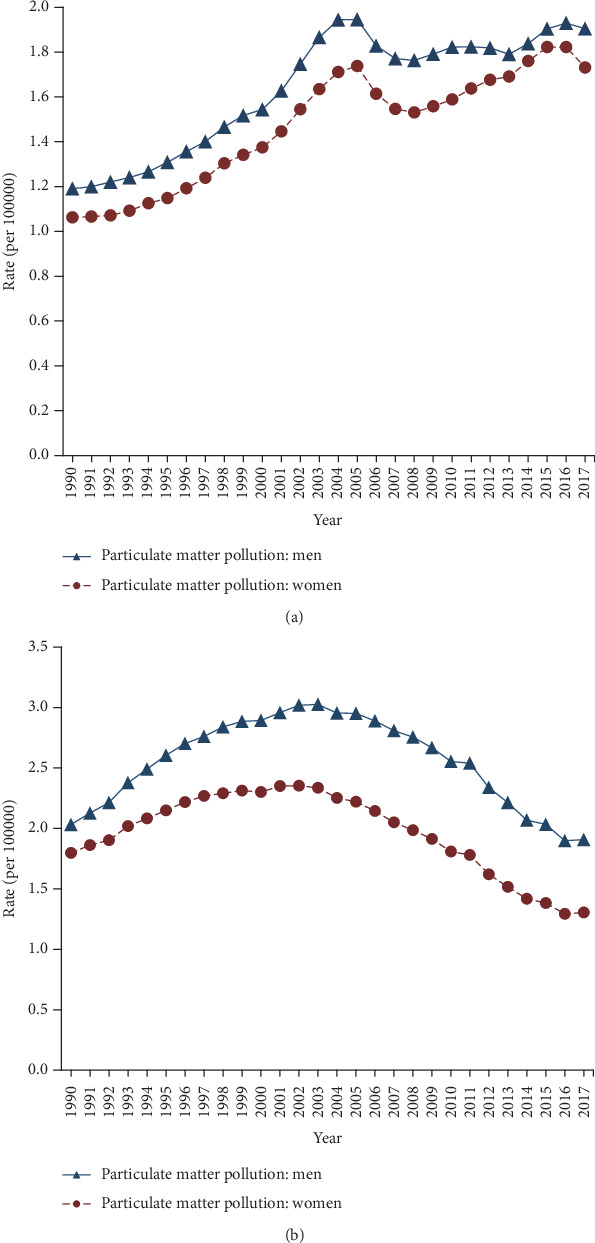
Trends in the age-standardized rates of type 2 diabetes attributable to particulate matter pollution in China and the U.S. from 1990-2017, at all ages. (a) China; (b) U.S.

Trends in age-standardized mortality rates of type 2 diabetes attributable to particulate matter pollution from 1990 to 2017 for China and U.S. are shown in Figure 2. The highest rate in China was 1.95/100,000 in men and 1.74/100,000 in women in 2005. In U.S., the highest rate was 3.03/100,000 in men in 2003 and 2.35/100,000 in women in 2002. The lowest rate in China was 1.19/100,000 in men and 1.06/100,000 in women in 1990, while in U.S., it was 1.91/100,000 in men and 1.30/100,000 in women in 2017. The U.S. showed a high attributable mortality, compared with China. In China, the mortality attributable to particulate matter pollution rapidly increased from 1990 to 2005 and subsequently decreased from 2005 to 2007 and almost increased since 2007 and began to decrease again in 2016 in China. In U.S., the mortality attributable to particulate matter pollution shows an increasing trend first and a decreasing trend almost since 2000. Overall, the age-standardized mortality rate of type 2 diabetes attributable to particulate matter pollution shows a general increasing trend in China while an increase before 2000 and subsequently a decreasing trend in the United States.

### 3.2. Age-Period-Cohort Analysis of Age-Standardized Mortality of Type 2 Diabetes Attributable to Particulate Matter Pollution

#### 3.2.1. Age Effect

The age effect showed an increasing trend for both sexes in both China and U.S. (see Figure 1(a)). Additionally, the age effect increased rapidly in China than U.S. From 25–29 to 90–94 age group, the RR of particulate-matter-pollution-attributable mortality increased by 136.65 times and 96.28 times in men and women in China, respectively; it increased by 288.13 times and 308.35 times in men and women in the U.S., respectively.

#### 3.2.2. Period Effect

A different pattern of period effect was observed between the two areas. The period effect showed a significantly increasing trend in China; while in the U.S., this study observed an increase from 1992 to 2007 and subsequently a tendency to stability from 2007 to 2017 (see Figure 1(b)). From 1992 to 2017, the RR of particulate-matter-pollution-attributable mortality increased by 3.01 times and 2.87 times in men and women in China, respectively; in the U.S., it generally increased by 2.12 times and 1.73 times in men and women, respectively.

#### 3.2.3. Cohort Effect

The cohort effect showed a decreasing trend in the two areas for both sexes (see Figure 1(c)). The relative risk of mortality attributable to particulate matter pollution peaked in the cohort born in 1902-1906. In China, from 1902–1906 birth cohort to the most recent birth cohort 1992-1996, RR of particulate-matter-pollution-attributable mortality decreased by 93.35% and 94.91% in men and women, respectively. In the U.S., RR of the mortality decreased by 92.26% and 91.20% in men and women, respectively.

## 4. Discussion

Diabetes is affected by biological factors, genetic factors, personal behavior, and environment factors. Particulate matter pollution is considered to be an increased risk for type 2 diabetes [8, 11, 13]. We performed a systematic comparison of trends of type 2 diabetes mortality attributable to particulate matter pollution in China and U.S. using the APC model, to explore the causes underlying the mortality trends and assess the effect of public health control policies.

The overall age-standardized mortality rate of type 2 diabetes attributable to particulate matter pollution varies from 1990 to 2017 in the U.S., and a significant decline was observed in recent years. This decline in U.S. was possibly associated with progress in diabetes care and management (as indicated by HbA1c levels less than 8%) [19] and tighter inpatient and outpatient glucose control [20] in recent years. Differently, a significant increase in the age-standardized mortality rates attributable to particulate matter pollution has been observed in China during the same period, which possibly indicates that the inadequate implementation of public health control policies, limited medical resources, and clinical treatment may have driven the increasing trend observed in the Chinese population. The age effect on type 2 diabetes mortality attributable to particulate matter pollution showed a generally increasing trend in both China and U.S., and the cohort effect showed a decreasing trend in the two areas. Interestingly, different period effects were observed in the two areas. An increasing trend was observed in China, while the period effect tended to be stable in the U.S. since 2007. Therefore, the three effects were discussed preliminarily in the following section.

### 4.1. Age Effect

The increasing age effect was observed in both China and U.S., which may suggest that as age increased, the risk of type 2 diabetes mortality attributable to particulate matter pollution increased. A previous study showed diabetes mortality increased steeply with advancing age [21], and this finding in our study was in line with the trend of diabetes mortality. In general, older people also have a higher risk to disease than younger people. The increasing age effect could be explained by the fact that the older patients may experience more exposure to low hygiene environments and less opportunities to effective measurements than those in the younger [22]. Moreover, diabetes complications, including cardiovascular disease, kidney disease, neuropathy, blindness, and lower-extremity amputation and comorbidities, are more frequent in old diabetics compared to their young counterparts [23–25]. All these factors may also impact the mortality of type 2 diabetes.

The age effect increased rapidly in China than U.S., which was also possibly attributable to that China elder population has high exposure to air pollution dominated by fine particulate matter (PM_2.5_) and ground level ozone (O_3_) [26, 27]. Apart from that, the population growth and China's aging [28] may also intensify this trend in China during the last decades; thus, more attention must be paid in the prevention and control of type 2 diabetes mortality for older people in China.

### 4.2. Period Effect

A different pattern of the period effect was observed in the two areas over the whole study periods. China showed a rapidly increasing trend, which indicated the period effect might be contributed to the increase in the linear trend of the mortality. However, in U.S., the period effect showed an increase from 1992 to 2007 and subsequently a tendency to stabilize from 2007 to 2017. As reported, the risk factors for type 2 diabetes include a strong family history of diabetes mellitus, age, overweight/obesity, and physical inactivity [29]. Air pollution mainly including particulate matter pollution has an increased association with the risk of type 2 diabetes [8–10]. The reason of the different period effects between the two areas was possibly related to increased exposure to air pollution mainly dominated by fine particulate matter (PM_2.5_) in China population [26, 27] while decreased exposure to air pollution in the U.S. in recent years [30]. Thus, with rapid development of economy and urbanization in China, which could lead to particulate matter air pollution, more measures should be made for prevention and control of air pollutant problem.

Diabetes is often accompanied by complications—such as cardiovascular disease, kidney disease, neuropathy, blindness, and lower-extremity amputation [23–25]. The U.S. has a progress in diabetes care and management [19] and tighter inpatient and outpatient glucose control [20] in recent years, while the rates of awareness, treatment, and control of diabetes are relatively low in China population [31]. In addition, type 2 diabetes is generally treated with drugs such as metformin hydrochloride. As for the oral antidiabetic drug therapy, the status in China is varied with a majority of type 2 diabetes patients altering their treatment regimen citing poor effectiveness [32]. Thus, China population similarly faces an increased risk of type 2 diabetes death while the risk began to be stable in the U.S. people.

### 4.3. Cohort Effect

The cohort effects declined continuously from the 1902-1906 birth cohort to more recent birth cohorts in the two areas, which indicated a decreased risk of the mortality in the younger generations. The decreased cohort effects observed in younger generations were usually possibly attributed to that younger generations may receive good education and have a strong awareness of health and prevention of disease [33], as the level of education was low in early stages and earlier birth cohorts had weak health awareness, so they realized neither the occurrence of diabetes nor the damage of obesity or overweight to human health. Because there has been occurrence of improvements in public health policy, treatment of medical conditions and implementation of diabetes screening in recent years; the more recent cohorts experienced a lower exposure to the diabetes risk, compared with the elder birth cohorts.

This study has some limitations. We only tried to bring forth scientific hypotheses regarding the causality of these trends of type 2 diabetes mortality attributable to particulate matter pollution in China and U.S., based on the available data and existing literatures. Despite that GBD mortality estimation incorporates methods to adjust for incomplete or missing vital registration (VR) and verbal autopsy (VA) data, general heterogeneity in data completeness and quality, and the redistribution of so-called garbage codes (insufficiently specific or implausible cause of death codes), there might be difficult to thoroughly avoid inaccuracy of data. Therefore, our results in the present study on trends of type 2 diabetes mortality should be treated carefully.

## 5. Conclusion

In summary, we evaluated the general trends in type 2 diabetes mortality attributable to particulate matter pollution in China and U.S. during the periods 1990-2017. The age-standardized morality rates attributable to particulate matter pollution are increasing in China, while the mortality in U.S. varies from China and a decline was observed in recent years. The age effect showed an increasing trend, and the cohort effect a declining trend in both China and U.S. Differently, the period effect showed an increasing trend in China while an increasing trend before 2007 and subsequently a tendency to stabilize in the U.S. Thus, the period effect may be the key factor affecting the increasing trend in China population. The population growth and China's aging will continuously drive type 2 diabetes mortality. It is necessary to educate the nation with the correct knowledge and adopting policies on particulate emission and techniques to reduce air pollution.

## Data Availability

The [The relative risks of type 2 diabetes mortality attributable to particulate matter pollution due to age, period and cohort effects] data used to support the findings of this study are included within the supplementary information file(s).

## Supplementary Materials

Supplementary Materials Supplementary Table A: goodness-of-fit in APC-C (APC models solved with constrained generalized liner model estimator) and APC-IE (age-period-cohort models solved with intrinsic estimator) models for mortality of diabetes attributable to particulate matter pollution. Supplementary Table B: diabetes mortality attributable to particulate matter pollution estimated coefficients for the age, period, and cohort effects. Supplementary Table C: the relative risks of diabetes mortality attributable to particulate matter pollution due to age, period, and cohort effects, China. Supplementary Table D: the relative risks of diabetes mortality attributable to particulate matter pollution due to age, period, and cohort effects, U.S.
