# Supplementary material for: Age-Period-Cohort Analysis of Type 2 Diabetes Mortality Attributable to Particulate Matter Pollution in China and the U.S
Source: J Diabetes Res. 2020 Jun 11;2020:1243947. doi: 10.1155/2020/1243947 (PMC7306083; doi:10.1155/2020/1243947)
Supplement: Supplementary Materials — Supplementary Table A: goodness-of-fit in APC-C (APC models solved with constrained generalized liner model estimator) and APC-IE (age-period-cohort models solved with intrinsic estimator) models for mortality of diabetes attributable to particulate matter pollution. Supplementary Table B: diabetes mortality attributable to particulate matter pollution estimated coefficients for the age, period, and cohort effects. Supplementary Table C: the relative risks of diabetes mortality attributable to particulate matter pollution due to age, period, and cohort effects, China. Supplementary Table D: the relative risks of diabetes mortality attributable to particulate matter pollution due to age, period, and cohort effects, U.S. [file 1243947.f1.docx]

Supplementary Table A: Goodness-of-fit in APC-C (APC models solved with constrained generalized liner model estimator), and APC-IE (age-period-cohort models solved with intrinsic estimator) models for mortality of diabetes attributable to particulate matter pollution.

| *df* | **APC-C** | **APC-IE** |
| --- | --- | --- |
|  | 81 | 48 |
| **U.S. Men** |  |  |
| Deviation | 72.04 | 0.48 |
| AIC | 4.19 | 4.12 |
| BIC | -286.86 | -212.20 |
| **U.S. Women** |  |  |
| Deviation | 54.84 | 0.32 |
| AIC | 3.74 | 3.88 |
| BIC | -304.06 | -212.36 |
| **China Men** |  |  |
| Deviation | 27.50 | 0.86 |
| AIC | 3.32 | 3.79 |
| BIC | -331.40 | -211.82 |
| **China Women** |  |  |
| Deviation | 68.45 | 1.37 |
| AIC | 3.63 | 3.61 |
| BIC | -290.44 | -211.31 |

AIC: Akaike Information Criterions; BIC: Bayesian Information Criterions.

Supplementary Table B: Diabetes mortality attributable to particulate matter pollution estimated coefficients for the age, period and cohort effects.

| **Factor** | **China (Coef.)** | | | **U.S. (Coef.)** | |
| --- | --- | --- | --- | --- | --- |
|  | **Men** | **Men** |  | **Men** | **Women** |
| **Age** |  |  | |  |  |
| 25–29 | -2.832383 | -2.574975 | | -3.910865 | -3.885235 |
| 30–34 | -2.148369 | -2.469505 | | -2.468373 | -2.425287 |
| 35–39 | -2.006912 | -2.292571 | | -2.01042 | -2.02243 |
| 40–44 | -1.470971 | -1.742863 | | -1.563684 | -1.566918 |
| 45–49 | -0.788008 | -1.020113 | | -0.3307195 | -0.3792326 |
| 50–54 | -0.424364 | -0.563774 | | 0.0797519 | 0.0906864 |
| 55–59 | -0.105933 | -0.101284 | | 0.4405964 | 0.4622649 |
| 60–64 | 0.3951401 | 0.6534926 | | 0.8875561 | 0.8619887 |
| 65–69 | 0.8140845 | 1.060502 | | 1.080008 | 1.049839 |
| 70–74 | 1.23995 | 1.505762 | | 1.280875 | 1.242234 |
| 75–79 | 1.476019 | 1.714886 | | 1.418797 | 1.378717 |
| 80–84 | 1.78603 | 1.861935 | | 1.606314 | 1.584205 |
| 85-89 | 1.98064 | 1.976256 | | 1.737617 | 1.763154 |
| 90-94 | 2.085076 | 1.992251 | | 1.752546 | 1.846014 |
| **Period** |  |  | |  |  |
| 1992 | -0.627632 | -0.592216 | | -0.5483907 | -0.4270379 |
| 1997 | -0.353122 | -0.326009 | | -0.1760328 | -0.1194629 |
| 2002 | 0.0153394 | 0.0129213 | | 0.0929492 | 0.1074238 |
| 2007 | 0.1797798 | 0.1192712 | | 0.2042866 | 0.167974 |
| 2012 | 0.3127497 | 0.3222021 | | 0.2258676 | 0.1510425 |
| 2017 | 0.4728845 | 0.4638305 | | 0.2013201 | 0.1200605 |
| **Cohort** |  |  | |  |  |
| 1902–1906 | 1.255024 | 1.188178 | | 1.432729 | 1.34121 |
| 1907–1911 | 1.128879 | 1.097585 | | 1.304136 | 1.275528 |
| 1912–1916 | 0.9805122 | 0.9524912 | | 1.171278 | 1.178393 |
| 1917–1921 | 0.8437071 | 0.8564259 | | 1.057293 | 1.085279 |
| 1922–1926 | 0.7362136 | 0.8071028 | | 0.896452 | 0.9543236 |
| 1927–1931 | 0.6336938 | 0.759579 | | 0.6907571 | 0.761866 |
| 1932–1936 | 0.4980802 | 0.6869488 | | 0.4523991 | 0.5452458 |
| 1937–1941 | 0.3661929 | 0.5669513 | | 0.2358445 | 0.3198801 |
| 1942–1946 | 0.1657029 | 0.3506603 | | 0.0401126 | 0.0659495 |
| 1947–1951 | 0.0510753 | 0.2307879 | | -0.1404443 | -0.1756161 |
| 1952–1956 | 0.038283 | 0.1846211 | | -0.3013201 | -0.4026935 |
| 1957–1961 | -0.141477 | -0.071065 | | -0.4222835 | -0.5536569 |
| 1962–1966 | -0.397639 | -0.418055 | | -0.5666616 | -0.676257 |
| 1967–1971 | -0.387981 | -0.418334 | | -0.7348553 | -0.7917167 |
| 1972–1976 | -0.678241 | -0.755687 | | -0.9034328 | -0.9148581 |
| 1977–1981 | -1.024793 | -1.16385 | | -0.9754785 | -0.9373169 |
| 1982–1986 | -1.247521 | -1.461573 | | -1.03784 | -0.9746488 |
| 1987–1991 | -1.363547 | -1.603977 | | -1.073259 | -1.012027 |
| 1992–1996 | -1.456165 | -1.78879 | | -1.125425 | -1.088885 |

Coef.: Coefficient.

Supplementary Table C: The relative risks of diabetes mortality attributable to particulate matter pollution due to age, period and cohort effects, China.

| **Factor** | **RR (95% CI)** | | | |
| --- | --- | --- | --- | --- |
|  | **Men** | **Women** | | |
| **Age** |  |  | | |
| 25–29 | 0.06 (0.00-2.42) | 0.08 (0.00-5.06) | | |
| 30–34 | 0.12 (0.01-1.18) | 0.08 (0.00-2.47) | | |
| 35–39 | 0.13 (0.02-1.03) | 0.10 (0.01-1.76) | | |
| 40–44 | 0.23 (0.05-1.08) | 0.18 (0.02-1.42) | | |
| 45–49 | 0.45 (0.14-1.46) | 0.36 (0.08-1.66) | | |
| 50–54 | 0.65 (0.24-1.75) | 0.57 (0.16-1.97) | | |
| 55–59 | 0.90 (0.39-2.06) | 0.90 (0.33-2.47) | | |
| 60–64 | 1.48 (0.77-2.85) | 1.92 (0.90-4.10) | | |
| 65–69 | 2.26 (1.35-3.78) | 2.89 (1.59-5.25) | | |
| 70–74 | 3.46 (2.27-5.25) | 4.51 (2.75-7.39) | | |
| 75–79 | 4.38 (2.97-6.44) | 5.56 (3.40-9.08) | | |
| 80–84 | 5.97 (3.91-9.10) | 6.44 (3.60-11.49) | | |
| 85-89 | 7.25 (4.33-12.13) | 7.22 (3.49-14.91) | | |
| 90-94 | 8.05 (4.24-15.26) | 7.33 (2.97-18.11) | | |
| **Period** |  |  |  |  |
| 1992 | 0.53 (0.34-0.84) | 0.55 (0.31-0.99) | | |
| 1997 | 0.70 (0.52-0.94) | 0.72 (0.50-1.05) | | |
| 2002 | 1.02 (0.85-1.21) | 1.01 (0.83-1.24) | | |
| 2007 | 1.20 (1.00-1.43) | 1.13 (0.92-1.38) | | |
| 2012 | 1.37 (1.03-1.82) | 1.38 (0.96-1.98) | | |
| 2017 | 1.60 (1.06-2.44) | 1.59 (0.91-2.77) | | |
| **Cohort** |  |  |  |  |
| 1902–1906 | 3.51 (1.36-9.06) | 3.28 (0.95-11.36) | | |
| 1907–1911 | 3.09 (1.41-6.78) | 3.00 (1.08-8.35) | | |
| 1912–1916 | 2.67 (1.38-5.16) | 2.59 (1.10-6.08) | | |
| 1917–1921 | 2.32 (1.32-4.10) | 2.35 (1.15-4.84) | | |
| 1922–1926 | 2.09 (1.25-3.49) | 2.24 (1.18-4.24) | | |
| 1927–1931 | 1.88 (1.14-3.12) | 2.14 (1.14-3.99) | | |
| 1932–1936 | 1.65 (0.94-2.89) | 1.99 (0.99-3.98) | | |
| 1937–1941 | 1.44 (0.75-2.79) | 1.76 (0.77-4.01) | | |
| 1942–1946 | 1.18 (0.54-2.59) | 1.42 (0.53-3.81) | | |
| 1947–1951 | 1.05 (0.42-2.66) | 1.26 (0.39-4.06) | | |
| 1952–1956 | 1.04 (0.36-3.04) | 1.20 (0.31-4.73) | | |
| 1957–1961 | 0.87 (0.25-3.02) | 0.93 (0.19-4.64) | | |
| 1962–1966 | 0.67 (0.16-2.87) | 0.66 (0.10-4.50) | | |
| 1967–1971 | 0.68 (0.14-3.33) | 0.66 (0.08-5.55) | | |
| 1972–1976 | 0.51 (0.07-3.51) | 0.47 (0.03-6.68) | | |
| 1977–1981 | 0.36 (0.03-4.98) | 0.31 (0.01-13.09) | | |
| 1982–1986 | 0.29 (0.01-10.03) | 0.23 (0.00-40.87) | | |
| 1987–1991 | 0.26 (0.00-28.07) | 0.20 (0.00-153.60) | | |
| 1992–1996 | 0.23 (0.00-2637.56) | 0.17 (0.00-16011.76) | | |

RR: Relative risk [RR = exp(coefficient)]; CI: Confidence interval; AIC: Akaike Information Criterions; BIC: Bayesian Information Criterions.

Supplementary Table D: The relative risks of diabetes mortality attributable to particulate matter pollution due to age, period and cohort effects, U.S.

| **Factor** | **RR (95% CI)** | | | |
| --- | --- | --- | --- | --- |
|  | **Men** | **Women** | | |
| **Age** |  |  | | |
| 25–29 | 0.02 (0.00-3.05) | 0.02 (0.00-5.82) | | |
| 30–34 | 0.08 (0.01-0.87) | 0.09 (0.01-1.24) | | |
| 35–39 | 0.13 (0.02-0.89) | 0.13 (0.01-1.22) | | |
| 40–44 | 0.21 (0.04-1.00) | 0.21 (0.03-1.31) | | |
| 45–49 | 0.72 (0.24-2.14) | 0.68 (0.19-2.47) | | |
| 50–54 | 1.08 (0.43-2.73) | 1.09 (0.37-3.20) | | |
| 55–59 | 1.55 (0.72-3.35) | 1.59 (0.65-3.86) | | |
| 60–64 | 2.43 (1.31-4.51) | 2.37 (1.16-4.83) | | |
| 65–69 | 2.94 (1.79-4.85) | 2.86 (1.61-5.06) | | |
| 70–74 | 3.60 (2.39-5.42) | 3.46 (2.17-5.53) | | |
| 75–79 | 4.13 (2.84-6.02) | 3.97 (2.58-6.11) | | |
| 80–84 | 4.98 (3.31-7.51) | 4.88 (3.04-7.81) | | |
| 85-89 | 5.68 (3.46-9.35) | 5.83 (3.28-10.36) | | |
| 90-94 | 5.77 (3.11-10.71) | 6.33 (3.10-12.96) | | |
| **Period** |  |  |  |  |
| 1992 | 0.58 (0.38-0.88) | 0.65 (0.40-1.06) | | |
| 1997 | 0.84 (0.64-1.10) | 0.89 (0.65-1.21) | | |
| 2002 | 1.10 (0.95-1.27) | 1.11 (0.84-1.31) | | |
| 2007 | 1.23 (1.05-1.43) | 1.18 (0.99-1.41) | | |
| 2012 | 1.25 (0.95-1.65) | 1.16 (0.85-1.60) | | |
| 2017 | 1.22 (0.81-1.85) | 1.13 (0.70-1.82) | | |
| **Cohort** |  |  |  |  |
| 1902–1906 | 4.19 (1.55-11.35) | 3.82 (1.21-12.07) | | |
| 1907–1911 | 3.68 (1.56-8.70) | 3.58 (1.32-9.69) | | |
| 1912–1916 | 3.23 (1.51-6.89) | 3.25 (1.35-7.84) | | |
| 1917–1921 | 2.88 (1.45-5.72) | 2.96 (1.33-6.58) | | |
| 1922–1926 | 2.45 (1.28-4.70) | 2.60 (1.22-5.54) | | |
| 1927–1931 | 2.00 (1.04-3.84) | 2.14 (1.00-4.59) | | |
| 1932–1936 | 1.57 (0.78-3.17) | 1.73 (0.76-3.90) | | |
| 1937–1941 | 1.27 (0.58-2.76) | 1.38 (0.56-3.41) | | |
| 1942–1946 | 1.04 (0.43-2.52) | 1.07 (0.38-2.99) | | |
| 1947–1951 | 0.87 (0.32-2.37) | 0.84 (0.26-2.70) | | |
| 1952–1956 | 0.74 (0.24-2.31) | 0.67 (0.18-2.53) | | |
| 1957–1961 | 0.66 (0.18-2.36) | 0.57 (0.13-2.58) | | |
| 1962–1966 | 0.57 (0.13-2.40) | 0.51 (0.09-2.77) | | |
| 1967–1971 | 0.48 (0.09-2.46) | 0.45 (0.07-3.09) | | |
| 1972–1976 | 0.41 (0.06-2.82) | 0.40 (0.04-3.94) | | |
| 1977–1981 | 0.38 (0.03-4.97) | 0.39 (0.02-7.86) | | |
| 1982–1986 | 0.35 (0.01-9.90) | 0.38 (0.01-17.73) | | |
| 1987–1991 | 0.34 (0.00-43.51) | 0.36 (0.00-92.95) | | |
| 1992–1996 | 0.32 (0.00-108565.61) | 0.34 (0.00-877620.90) | | |

RR: Relative risk [RR = exp(coefficient)]; CI: Confidence interval; AIC: Akaike Information Criterions; BIC: Bayesian Information Criterions.
